# Mechanical Properties, Hydration Mechanisms, and Microwave-Absorbing Properties of Alkali-Activated Blast-Furnace Slag Containing Steel Slag

**DOI:** 10.3390/ma19132761

**Published:** 2026-06-29

**Authors:** Qian Wang, Xiaotong Peng, Yuxin He, Zhenhua Yang, Ziqi Li, Yulin Wang, Taibing Wei, Rong Wang, Huawei Li

**Affiliations:** 1 School of Civil Engineering and Architecture, Wuyi University, Wuyishan 354300, China; wangqian@wuyiu.edu.cn (Q.W.); wangrong@wuyiu.edu.cn (R.W.); 2 School of Civil and Transportation Engineering, Guangdong University of Technology, Guangzhou 510006, China

**Keywords:** alkali-activated materials, steel slag, mechanical properties, hydration mechanisms, microwave-absorbing properties

## Abstract

As a novel low-carbon material, alkali-activated materials (AAMs) can effectively mitigate the environmental burden caused by the cement industry, and their functional development can further enhance their additional commercial benefits. This study employed alkali-activated blast-furnace slag (AAS) as a matrix and incorporated steel slag (SS) as a functional component, and the compressive strength, workability, shrinkage characteristics, microstructure, and microwave-absorbing properties of SS-containing AAS were systematically investigated. The results show that although the low reactivity of SS impairs the compressive strength of AAS, it effectively reduces the setting rate of AAS. At an SS dosage of 50% (sample B-S50), the 28-day drying shrinkage of AAS reached a minimum value of 778 με. The dissolution and hydration of SS provide additional Ca^2+^ and OH^−^ for AAS, thereby effectively promoting the hydration of blast-furnace slag and facilitating the formation of C–(A)–S–H and N–A–S–H gels. Moreover, SS acts as a conductive functional component, enhancing the conductivity of AAS and enabling a minimum reflection loss of −29.47 dB with 0.53 GHz effective bandwidth at 20 mm thickness. After further modification with steel fibers, the thickness-dependence of the microwave-absorbing properties of AAS was reduced, allowing effective absorption across multiple thicknesses (5 mm, 15 mm, and 25 mm). This study offers new insights into the high-value utilization of low-reactivity industrial solid waste and offers design methods for its functional development.

## 1. Introduction

As global industrialization and urbanization accelerate, there is a continuous growth in the demand for construction materials. Cement, as the most critical material in civil engineering, is produced on a massive scale, leading to severe resource depletion and environmental pollution [1,2,3]. Statistics indicate that the cement industry contributes approximately 5–8% of global CO_2_ emissions [4,5], and its carbon footprint has become a pressing issue, positioning it as a key point of breakthrough for achieving carbon neutrality in the construction sector [6]. To reduce the dependence on cement and lower its consumption, the development of alternative cementitious binders is urgently needed. Alkali-activated materials (AAMs), a novel type of green cementitious material, offer significant low-carbon characteristics and environmental advantages, aligning with the Sustainable Development Goals and representing an ideal substitute for traditional cementitious binders [7,8]. Compared to ordinary Portland cement, AAMs can reduce carbon emissions by about 60%, promoting the recycling of industrial solid waste, and yield remarkable environmental and economic benefits [9,10]. Therefore, the development of AAMs is of great significance for promoting green and low-carbon transformation in the construction industry and advancing sustainable development.

Alkali-activated slag (AAS) is a common low-carbon, environmentally friendly type of AAM. Owing to its unique mechanical advantages and environmental benefits, it is regarded as a preferred precursor for producing AAMs and is widely used in civil engineering [9,11]. AAS primarily uses aluminum- and silicon-rich blast furnace slag (BFS) as a precursor. Under the action of an alkali activator, dissolution–polycondensation reactions occur, forming a three-dimensional network gel structure composed of tetrahedral [SiO_4_]^4−^ and [AlO_4_]^5−^ as fundamental building units. Its dense microstructure and the absence of calcium hydroxide in the hydrates endow AAS with excellent mechanical properties and durability [2,12]. However, the use of strongly alkaline activators leads to excessively rapid early reaction kinetics, which adversely affect the workability and volume stability of the material and severely limit its engineering applications [1,13]. To address this issue, some studies suggest that incorporating low-reactivity mineral admixtures into AAS can alleviate the excessively fast early hydration reaction and the associated large early-age matrix shrinkage, without significantly compromising mechanical properties and durability [14,15]. This approach is expected to become an effective approach for improving the performance of AAS.

Steel slag (SS) is a typical solid waste generated in the course of steel production, yet its low reactivity results in a relatively low utilization rate [1]. Nevertheless, due to the presence of certain chemical components similar to those of Portland cement, such as CaO, SiO_2_, and Al_2_O_3_, SS possesses a certain potential for cementitious activity [16]. The free CaO and MgO present in SS may cause volume instability, generating expansion stresses that can readily damage the cementitious matrix. Guo et al. [6] explored the influence of SS on the performance of AAMs and found that SS promotes the refinement of the pore structure and improves workability. Similarly, Rudić et al. [17] and Li et al. [1] further confirmed the inhibitory effect of SS on shrinkage deformation during the hydration of cementitious materials. For some common cementitious systems with rapid setting rates, including alkali-activated systems, sulfoaluminate cement systems, and high-alumina cement systems, there exist problems such as fast setting and hardening, as well as insufficient workable time. These issues can be regulated by taking advantage of the low hydration activity of SS. Therefore, introducing SS into AAS is a feasible approach to balance its setting characteristics and volume stability.

The functional development of AAS is of great significance for enhancing its added commercial value. SS contains a certain amount of Fe-bearing phases, such as Fe_2_O_3_ and Fe_3_O_4_, which are generally considered to possess a remarkable electromagnetic loss capability. Recent studies have reported that SS can serve as a functional filler, endowing cementitious materials with microwave-absorbing properties [18,19]. Bai et al. [18] reported that a 15% SS dosage increased the compressive strength of foamed concrete by 18.59% and enhanced the conductivity by 37.87% compared to the control group, while also facilitating the formation of conductive pathways in the cementitious matrix. This finding was further corroborated by Meng et al. [20]. Moreover, Dai et al. [21] investigated the microwave-absorbing properties of SS-containing cementitious materials and demonstrated that the iron-bearing components in SS can effectively modulate the electromagnetic parameters, enabling the SS-containing cementitious materials to achieve a minimum reflection loss of −21 dB. Collectively, these studies fully illustrate the potential of SS in improving the electrical and microwave-absorbing properties of construction materials. However, the microwave absorption properties of AAS containing SS have not been fully studied, and the intrinsic mechanisms underlying its electromagnetic loss remain unclear. Therefore, while optimizing the fundamental mechanical properties and workability of AAS, how to further enhance its microwave-absorbing performance constitutes an urgent research task.

This study takes AAS as the matrix and modifies its performance by adjusting the dosage of SS, aiming to develop AAS with satisfactory mechanical properties, workability, and microwave-absorbing properties. The influence of SS on the compressive strength, workability, shrinkage characteristics, microstructure, and microwave-absorbing properties of AAS were systematically investigated. Furthermore, steel fibers (SFs) were introduced as absorbers to further enhance the electromagnetic loss capacity of AAS, thereby effectively enhancing its microwave-absorbing properties. This study seeks to develop an eco-friendly microwave-absorbing material that combines good mechanical properties with microwave absorption functionality. These findings not only contribute to increasing the high-value utilization of industrial solid waste but also offer new design insights for the functional design of alkali-activated materials.

## 2. Materials and Test Methods

### 2.1. Materials

**Figure 1:**
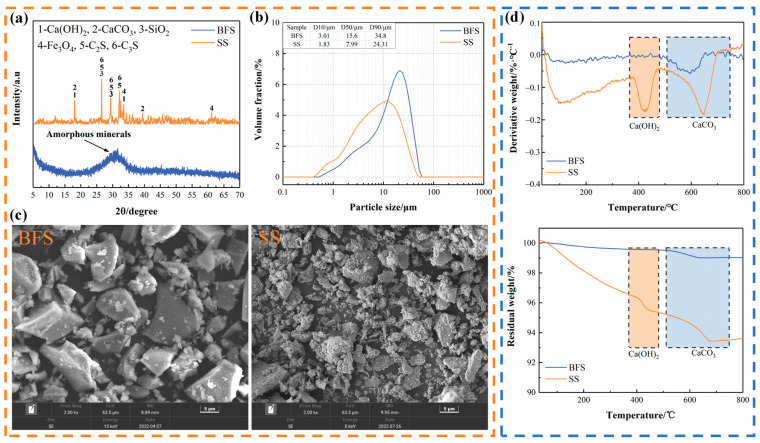
Basic performance of BFS and SS: (**a**) mineral compositions; (**b**) particle-size distributions; (**c**) microstructure; and (**d**) TG/DTG curves.

The materials used in this study include BFS and SS, which were supplied from Wuhan Vcem Intelligent Technology Co., Ltd. (Wuhan, China). The Blaine and specific gravity of BFS were 440 m^2^·kg^−1^ and 2.9 g·cm^−3^, respectively, and these of SS were 408 m^2^·kg^−1^ and 3.68 g·cm^−3^. The chemical and mineral compositions of BFS and SS were listed in Table 1 and Figure 1a, respectively. Figure 1b represents the particle-size distribution of BFS and SS, as acquired by a laser particle-size analyzer (Mastersizer 2000, Malvern Panalytical Ltd., Malvern, UK). The result shows that the median diameter (D50) of SS (7.99 μm) is smaller than that of BFS (15.6 μm). The microstructures of BFS and SS were characterized by scanning electron microscopy (SEM), as shown in Figure 1c. The SEM image shows that the BFS and SS particles are both subangular to angular in shape, and the particle size of SS is smaller than that of BFS, which is consistent with the results of particle size distribution. The weight loss of the two materials were analyzed by thermal gravimetric (TG) and differential thermogravimetry (DTG, STA449F5) methods, with the results shown in Figure 1d.

To obtain the alkaline solution, commercial water glass (with 3.3 modulus and 35% solid content) and sodium hydroxide were adopted. According to previous literature [22], the modulus of water glass (SiO_2_/Na_2_O molar ratio) was adjusted by adding sodium hydroxide to increase the number of Na_2_O. Based on previous studies [23], water glass (modulus 1.6) has been demonstrated to improve the performance of AAS. Consequently, this study sets the target modulus of water glass at 1.6. Then, the modified water glass solution with 1.6 modulus was obtained according to Equation (1).

(1) $$m=0.361\times (3.3-M_{a})/3{.3M}_{a}$$

where m (g) is the mass of sodium hydroxide added per gram of water glass solution; 3.3 is the initial modulus of the water glass; and M_a_ is the target modulus of alkaline solution.

### 2.2. Preparation of Samples

To investigate the impact of SS dosage on the properties of AAS, different SS dosages (0%, 10%, 30%, 50%, 70%, 90%) were used to replace BFS. The modified water glass (1.6 modulus) was used as alkaline activator. The liquid-to-binder ratio and water-to-solid ratio of the AAS mortar were 0.55 and 0.35, respectively. To mitigate the substantial heat generated by the alkaline solution during the mixing process, it is prepared at least 24 h in advance. The detailed mix design of AAS mortars is shown in Table 2.

After mixing, the mortar sample was poured into steel molds (40 × 40 × 40 mm^3^ for compressive strength test, and 40 × 40 × 160 mm^3^ for resistivity test), and cured in a room (20 °C and 95% RH). The mix proportions for the paste sample are consistent with that of the mortar, with the only difference being the absence of sand. The paste samples are cured in the same manner in the standard curing room until they reach a specified age, after which anhydrous ethanol is used to terminate the hydration process. The paste samples were then broken into small blocks using a small hammer and subjected to vacuum drying at 60 °C for 2 h. Some block samples were retained, while other blocks were ground to pass through a 75 μm standard sieve for X-ray diffraction (XRD), TG/DTG, and SEM.

### 2.3. Testing Procedures

#### 2.3.1. Mechanical Properties

The compressive strength test of all the samples cured for 3 d and 28 d was conducted by using a compressive testing machine (DYE-300, Tianjin Gangyuan Test Instrument Co., Ltd., Tianjin, China) based on Chinese standard GB/T 17671-2021 [24]. The loading speed of the compressive testing machine was set as 2.4 kN/s. Three parallel samples of each mixture were tested, and the average value and standard deviation were calculated.

#### 2.3.2. Workability Test

The fluidity was tested following the requirements of Chinese standard GB/T 2419-2005 [25]. Following 25 vibrations on the flow table, the spread diameter were measured to calculate the value of fluidity of samples. The setting time for each mixture was tested based on Chinese standard JGJ/T 70-2009 [26], with the initial setting time recorded upon the mortar strength reaching 0.3 MPa and the final setting time noted when the strength reached 0.7 MPa.

#### 2.3.3. Drying Shrinkage Test

The drying shrinkage test was carried out following the Chinese standard JGJ/T 70-2009 using a digital shrinkage meter. The mortar samples were demolded after curing in the standard curing room (20 °C and 95% RH) for 7 d. The initial length of specimen was recorded after calibrating the micrometer on the comparator by standard rod. Then, the samples were sent to the thermostatic chamber (20 °C and 65% relative humidity) for further curing. Subsequently, the length of samples was recorded on days 1, 3, 7, 14, 21, and 28, respectively. Three samples of each mixture were tested, and the shrinkage values were calculated using Equation (2).

(2) $$\varepsilon =(L_{0}-L_{t})/(L-L_{d})$$

where ε is the drying shrinkage at the corresponding curing day; *L*_0_ is the initial length of the mortar sample; *L_t_* is the length of the mortar sample at the corresponding curing day; *L* is the length of mortar sample (160 mm); and *L_d_* is the sum of the lengths of two shrinkage copper nails buried in the mortar sample (20 mm ± 2 mm).

#### 2.3.4. Microstructural Analysis

The hydrates phase compositions were analyzed using an X-ray diffractometer (Bruker, D8 Advanced, Karlsruhe, Germany), with a scanning range of 5–70° (2*θ*) and a scanning rate of 2°/min. The caloric effects and weight changes of hydrates were characterized using a thermogravimetric analyzer (NETZSCH, STA449F5, Selb, Germany). Powder samples prepared from the paste were heated at 10 °C/min from ambient temperature to 800 °C in an alumina crucible under a nitrogen atmosphere. The microstructures of block samples were examined by SEM (Hitachi, SU8600, Tokyo, Japan) equipped with an integrated EDS device, with the acceleration voltage maintained at 15 kV. To ensure good surface conductivity, the exposed fracture surface was coated with gold sputtering using an ion-sputtering instrument.

#### 2.3.5. Electrical Resistivity

**Figure 2:**
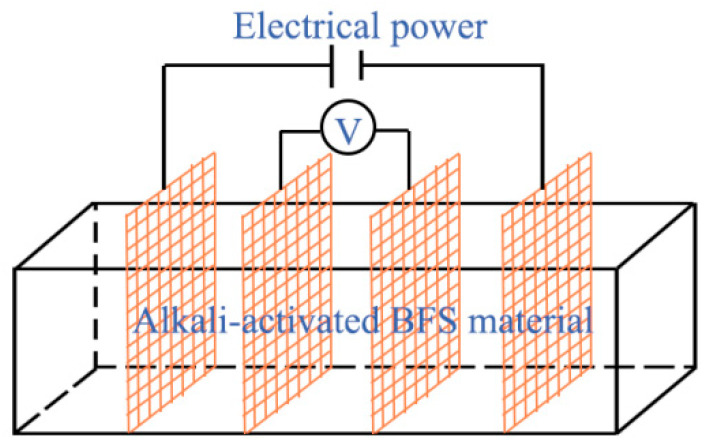
Schematic diagram of electrical resistivity test.

The resistivity of the samples at 28 d was determined by employing the four-probe method. Figure 2 shows the schematic diagram of electrical resistivity test. Four copper electrodes (30 × 40 mm^2^) were vertically inserted into the mortar sample at equal intervals. A digital multimeter and a DC power supply were used to construct the external circuit connections, and the voltage and current values were recorded to calculate the electrical resistivity. The equation for resistivity is as follows:

(3) $$\rho =\frac{U\cdot S}{I\cdot L}$$

where ρ is the electrical resistivity value; *U* and *I* are the voltage and current values recorded by voltmeter and ammeter; *S* is the contact area between the copper electrode and AAS mortar; and *L* is the average distance of the probes.

#### 2.3.6. Microwave-Absorbing Properties

**Figure 3:**
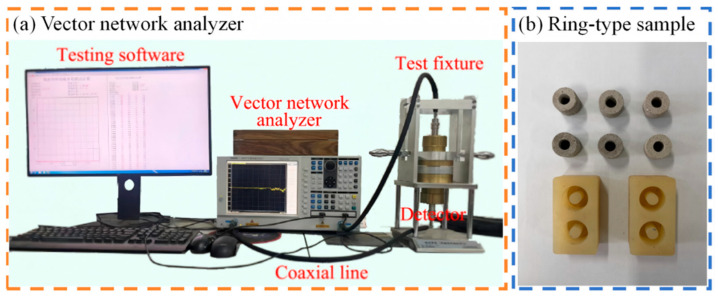
Experimental setup of microwave-absorbing properties.

According to Chinese Standard GJB 2038A-2011 [27], the electromagnetic parameters of mortar sample were analyzed using a vector network analyzer with the coaxial transmission method in the range of 0.1 to 5 GHz. It should be noted that the operating frequencies of currently commonly used communication devices, such as FM broadcasting, 3G, 4G, and 5G communication systems, are mainly concentrated in the range of 0.1–5 GHz. In view of this, this study investigates the electromagnetic wave absorption performance within this frequency band. The experimental setup is illustrated in Figure 3. The ring-type sample with 10.06 mm inner diameter, 23.15 mm outer diameter, and 20.00 mm height was used for this test. The reflection loss (RL) was subsequently calculated on the basis of transmission line theory.

## 3. Results and Discussion

### 3.1. Compressive Strength

**Figure 4:**
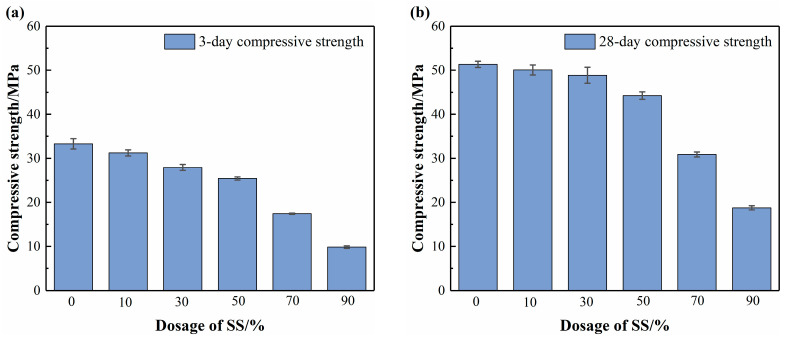
Compressive strength of AAS with different SS dosages: (**a**) 3 d; and (**b**) 28 d.

Figure 4 presents the 3 d and 28 d compressive strengths of AAS containing varying dosages of SS. Overall, both the 3 d and 28 d compressive strengths of AAS exhibited a decreasing trend with increasing SS dosages. As the SS dosages were less than 50%, the reduction in compressive strength was relatively low. Specifically, B-S0, B-S10, and B-S30 exhibited 3 d strengths of 33.3, 31.2, and 27.9 MPa, which increased to 51.3, 50.1, and 48.9 MPa at 28 d, respectively. This suggests that at low SS contents, SS remains hydraulically active at later stages, effectively maintaining strength development. However, as the dosage exceeded 50%, a more pronounced decline was observed. At 3 days, the compressive strength reductions of AAS were 6.2%, 16.1%, 23.6%, 47.6%, and 70.4% with increasing SS dosage. These values decreased at 28 days to 2.5%, 4.8%, 13.8%, 39.9%, and 63.4%, respectively. This can be mainly attributed to the reactivity of SS, which mainly manifests in the later hydration stage, yet its overall hydration activity remains significantly lower than that of BFS, thereby retarding the strength development [3]. According to You et al. [28], mineral phases such as C_3_S and C_2_S in SS are the key components promoting the reaction in the AAS matrix, producing hydrates similar to those of Portland cement. Consequently, the early strength development of AAS mainly originates from the complexation reaction between BFS and the alkaline activator, while the SS mineral phases gradually react at later stages, thereby mitigating the decline in later-age strength.

### 3.2. Workability

**Figure 5:**
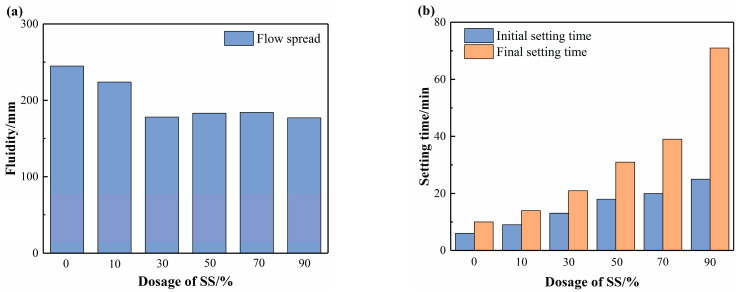
Workability of AAS with different SS dosages: (**a**) fluidity; and (**b**) initial and final setting times.

Workability is one of the key indicators for the engineering application of AAS. Figure 5 shows the variations in the fluidity and setting time of the AAS matrix with different SS dosages. As indicated in Figure 5a, the fluidity of AAS initially decreases continuously with increasing SS dosage and then tends to stabilize. Guo et al. [6] indicated that the low reactivity and low water demand of SS contribute to improving the fluidity of AAS. However, similar results were not observed in this study. This may be attributed to the larger particle size of SS compared to BFS, as the resulting higher specific surface area tends to increase the water demand of AAS, thereby reducing its fluidity.

Figure 5b shows that the setting times of AAS increase significantly with increasing SS dosage, and the increasing rate becomes more pronounced at higher SS dosages. This is attributed to the low early-age reactivity of SS, and the resulting dilution effect caused by replacing BFS with SS reduces the early hydration rate of AAS, thereby markedly prolonging its setting time. Additionally, Li et al. [1] suggested that a higher SiO_2_/Al_2_O_3_ ratio of precursor material leads to a longer setting time of AAMs. In this study, the SiO_2_/Al_2_O_3_ ratios of BFS and SS were 1.36 and 4.49, respectively, which is consistent with their findings. Notably, the rapid setting characteristic of AAS is a major factor limiting its engineering application, and the use of SS can effectively address this issue. Mohamed et al. [29] reported that the initial setting time of AAS varied between 8 and 24 min depending on the molar concentration of sodium silicate and sodium hydroxide solutions. Although the AAS prepared in this study still does not meet the typical setting time requirements for common cementitious materials (initial setting ≤ 45 min; final setting ≥ 360 min), the approach of incorporating low-reactivity materials provides an important design approach for regulating the setting time of AAS.

### 3.3. Drying Shrinkage

**Figure 6:**
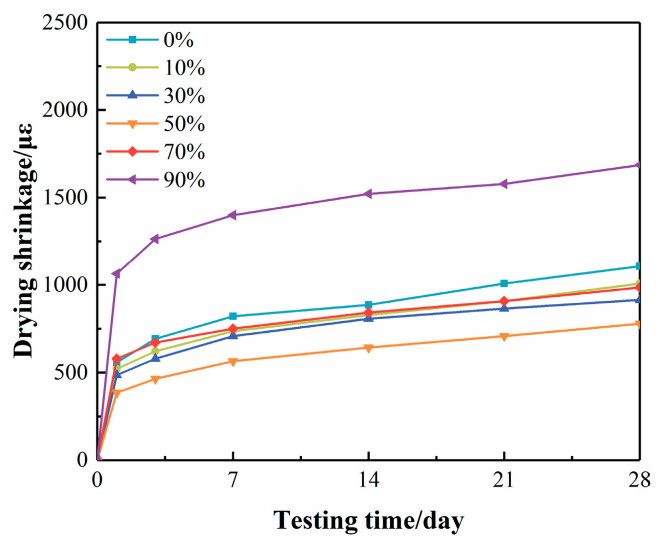
Drying shrinkage of AAS with different SS dosages.

Figure 6 shows the evolution of drying shrinkage of AAS within 28 days. The results indicate that the drying shrinkage increased rapidly within the first 3 days. As the time progressed, the increasing rate gradually slowed down. As the SS dosage was less than 50%, the increase in dosage led to a reduction in the total drying shrinkage at 28 days, reaching a minimum value of 778 με (sample B-S50). Subsequently, as the SS dosage further increased to 70% and 90%, the drying shrinkage increased significantly to 986 με and 1686 με, respectively. According to previous literature [30,31], drying shrinkage is primarily attributed to changes in capillary pressure resulting from the evaporation of free water. The appropriate dosage of SS can help improve the pore structure of the AAS system (since the particle size of SS is smaller than that of BFS), partially blocking the connected pores, thereby lowering the capillary pressure generated during the drying process. However, as the SS dosage continues to increase, the drawback of the low early-age reactivity of SS becomes dominant, leading to a reduced amount of hydrates and a decreased ability of the matrix to resist capillary pressure.

Previous research indicated that effective strategies to mitigate drying shrinkage have typically relied on the incorporation of chemical admixtures, fiber materials, and the optimization of curing regimes [32,33]. However, the use of low-reactivity or even inert fillers in AAS not only expands the source of precursor materials but also provides a feasible approach to improving its volume stability, which is of positive significance for promoting the cost-effective and sustainable application of AAMs.

### 3.4. XRD Analysis

**Figure 7:**
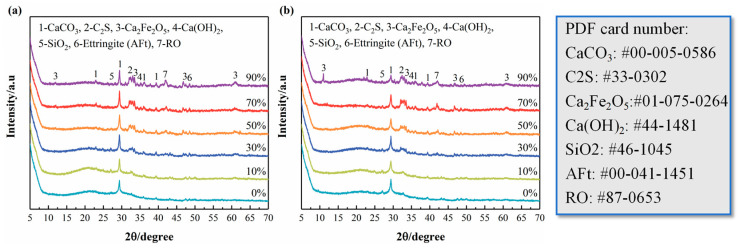
XRD pattern of AAS with different SS dosages: (**a**) 3 d; and (**b**) 28 d.

Figure 7 illustrates the XRD patterns of AAS at 3 and 28 days, with nearly identical hydrate diffraction signals detected. This indicates that the compositions of hydrates did not change with increasing curing age. The hydrates mainly include ettringite (AFt), SiO_2_, C_2_S, RO, CaCO_3_, and Ca(OH)_2_.

At 3 days, as the SS dosage increased, the peak intensities of C_2_S and RO phase increased. These low-reactivity mineral phases mainly originate from SS (see Table 1), and their presence is generally considered to reduce the reaction rate of AAS. The peaks of SiO_2_ were observed when the SS dosage was less than 50%. This was mainly related to the high SiO_2_ content in BFS. Notably, when the SS dosage was relatively high (exceeding 30%), the AFt peaks were observed. This can be attributed to the additional Ca^2+^ provided by f-CaO in SS, which creates favorable conditions for AFt formation. The hydration of SS can generate Ca(OH)_2_. However, in the XRD patterns of AAS with a low SS dosage, no obvious characteristic peaks of Ca(OH)_2_ were detected. Only when the SS dosage exceeded 50% were weak Ca(OH)_2_ peaks observed. This result indicates that BFS consumes the Ca(OH)_2_ generated by the hydration of SS during the reaction, suggesting a possible synergistic hydration effect between SS and BFS.

At 28 days, a distinct C_2_S peak was still observable, and its peak intensity gradually increased with increasing SS dosage. This is mainly due to the C_2_S mineral phase in SS having a high crystallinity and low hydration activity, leading to its slower reaction during the hydration process [34]. The later-age compressive strength of AAS mainly derives from gel-like hydrates. Given that the alkali activator used in this study is sodium silicate, it can be inferred that the gels in the AAS are primarily C–(A)–S–H and N–A–S–H. However, since these amorphous hydrates cannot be detected by XRD, the content of gel-like hydrates in SS-containing AAS will be further revealed by TG analysis.

### 3.5. TG-DTG Analysis

**Figure 8:**
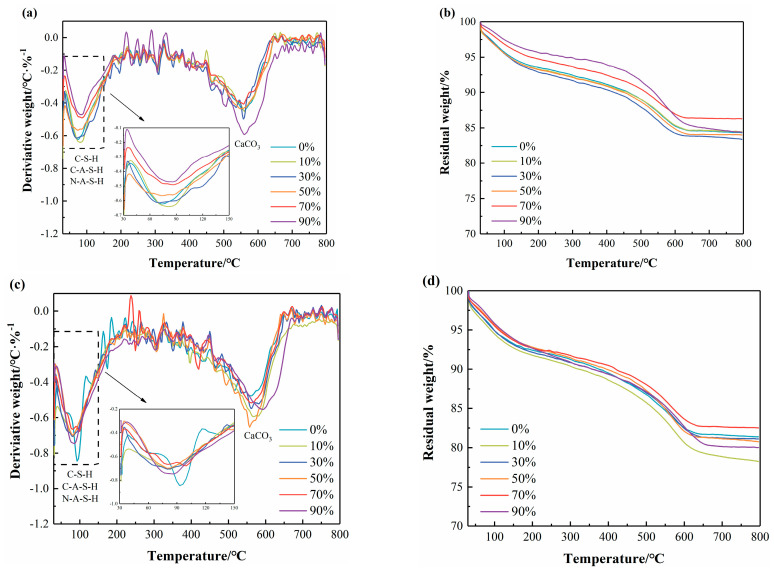
TG/DTG curve of AAS with different SS dosages: (**a**) and (**b**) for 3 d; and (**c**) and (**d**) for 28 d.

Figure 8 displays the TG/DTG curves of AAS at 3 and 28 days. The DTG curves reveal two distinct peaks in the ranges of 30–150 °C and 500–750 °C. The weight loss in the 30–150 °C range mainly corresponds to the loss of bound water from C–(A)–S–H and N–A–S–H in AAS, while the weight loss in the 500–750 °C range is attributed to the decomposition of CaCO_3_ with different crystallinities in AAS [35]. For ease of comparison, Table 3 lists the weight loss percentages of hydrates in these two specific temperature ranges.

C–(A)–S–H and N–A–S–H are considered the main hydrates of AAMs using a calcium-rich precursor, playing a crucial role in strength development. At 3 days, with increasing SS dosage, the amounts of C–(A)–S–H and N–A–S–H first increased and then gradually decreased. Generally, low-reactivity SS as a precursor is not conducive to promoting the formation of gel-like hydrates. However, a low SS dosage increased the weight loss in the 30–150 °C range, which may be related to the synergistic hydration effect between BFS and SS. The Ca^2+^ and OH^–^ provided by SS are beneficial for the hydration of BFS, promoting the formation of C–(A)–S–H. This observation confirms the speculation from the XRD analysis. At 28 days, the weight loss in the 30–150 °C range for all specimens generally maintained a similar trend to that at 3 days, but the group with the highest amounts of C–(A)–S–H and N–A–S–H corresponded to an SS dosage of 10%. This result indicates that the synergistic hydration effect between BFS and SS still functions at 28 days, albeit slightly weaker than at 3 days. At high SS content (exceeding 50%), the dilution effect becomes dominant, markedly reducing the amount of hydration products. This diminishes the otherwise achievable synergistic hydration effect between SS and BFS, ultimately lowering the strength. Notably, a considerable proportion of CaCO_3_ was detected in all samples, which mainly originated from the carbonation of AAS paste during curing and drying, as well as from SS (see Figure 1d).

### 3.6. SEM/EDS Analysis

**Figure 9:**
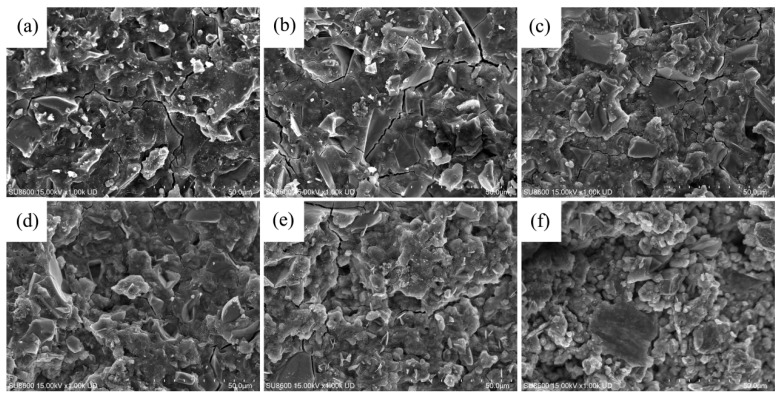
Microstructure of AAS with different SS dosages at 3 d: (**a**) 0%; (**b**) 10%; (**c**) 30%; (**d**) 50%; (**e**) 70%; and (**f**) 90%.

**Figure 10:**
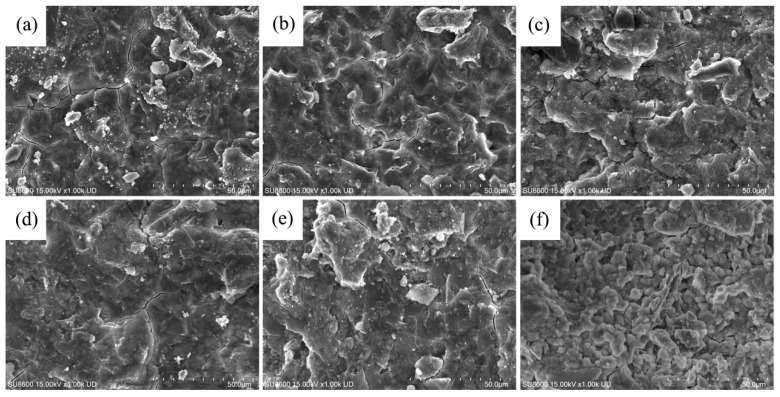
Microstructure of AAS with different SS dosages at 28 d: (**a**) 0%; (**b**) 10%; (**c**) 30%; (**d**) 50%; (**e**) 70%; and (**f**) 90%.

Figure 9 and Figure 10 show the SEM images of AAS at 3 and 28 days. As the SS dosage was less than 50%, a large amount of gel structure was observed. These gels were uniformly distributed and tightly connected, forming the early-age structural skeleton of AAS. A further increase in SS dosage leads to a decrease in the amount of gel hydrates, and more pores appear among the gel network, leading to a significant reduction in the overall denseness of the microstructure. Some unhydrated SS particles were also observed. This is mainly because a high SS dosage reduces the hydration degree, and the fewer gel hydrates weaken the binding effect between the structural skeletons, which constitutes the main cause for the reduced compressive strength at high SS replacement levels. Notably, as the SS dosage was more than 50%, obvious micro-cracks were observed in the hydrated structure of AAS. This is mainly due to the rapid reaction promoted by the strong alkali activator, which caused thermal stress within the system, leading to the formation of numerous cracks in the hydrates. With increasing SS dosage, the micro-cracks in the AAS hydrated structure decreased. This can be attributed to the regulating effect of SS: SS delayed the overall reaction rate, reducing the accumulation of early hydration heat; its slight expansion characteristics helped fill and compensate for micro-cracks, thereby improving the structural compactness.

**Figure 11:**
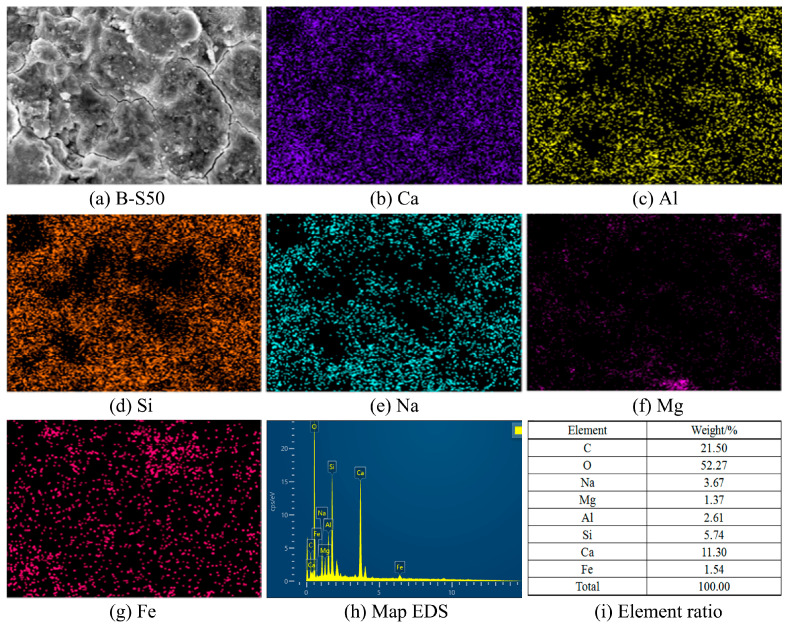
EDS analysis of B-S50.

Figure 11 shows the EDS results of sample B-S50. It can be seen that the chemical composition of hydrates in SS-containing AAS is Ca, Al, Si, Na, Mg, and Fe. Among these, Ca, Al, Si, and Na mainly originate from C–(A)–S–H and N–A–S–H. Mg is primarily associated with the MgO in SS and may participate in hydration to form M–A–S–H. Additionally, the Fe detected in the hydrates was 1.54%, indicating that Fe may also participate in the reaction of AAS, forming Fe-doped hydrates. Recent studies have shown that although Fe-bearing mineral phases have low reactivity, they may participate in the gel reaction to form Fe–C–S–H or sodium iron silicate hydrate [36,37]. However, the formation of these hydrates may require further verification using additional testing methods.

### 3.7. Microwave-Absorbing Properties and Mechanisms

#### 3.7.1. Electromagnetic Parameter Analysis

**Figure 12:**
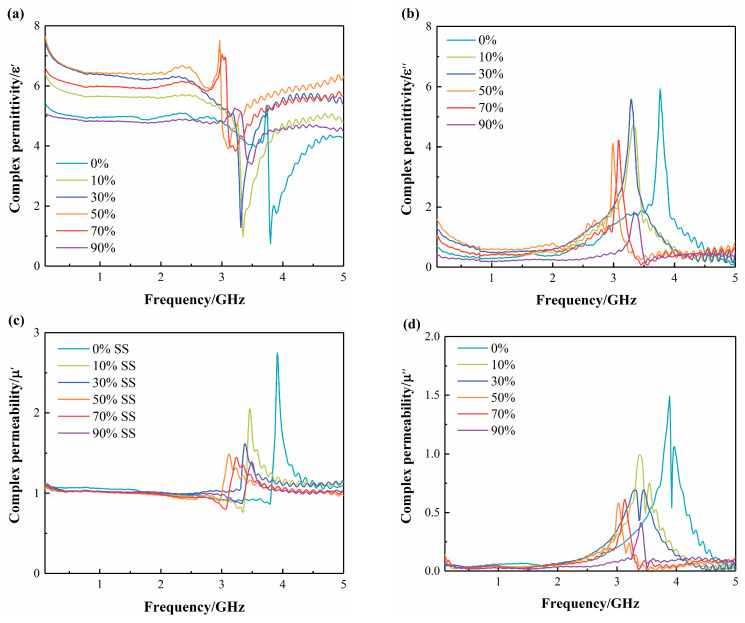
Electromagnetic parameters of AAS with different SS dosages (**a**) $\varepsilon^{\prime }$; (**b**) $\varepsilon^{\prime \prime }$; (**c**) $\mu^{\prime }$; (**d**) $\mu^{\prime \prime }$.

Electromagnetic parameters are critical indicators that reflect the polarization response and energy dissipation capability of a material under alternating electric and magnetic fields. Specifically, they refer to the permittivity and permeability, whose magnitudes are closely related to microwave-absorbing properties. Among these, the real parts of permittivity ($\varepsilon^{\prime }$) and permeability ($\mu^{\prime }$) reflect the ability of materials to store electric energy and magnetic energy, while the imaginary parts ($\varepsilon^{\prime \prime }$ and $\mu^{\prime \prime }$) correspond to its dielectric loss and magnetic loss capacities, respectively. In this section, the four basic electromagnetic parameters of all specimens are presented, which can be directly used to evaluate the dielectric loss tangent and magnetic loss tangent of the prepared materials. Figure 12 presents the electromagnetic parameters of AAS. It is evident from the figure that the real and imaginary parts of permittivity and permeability of SS-containing AAS exhibit frequency-dependent variations to different extents. However, the B-S0 displays relatively high $\varepsilon^{\prime }$ and $\mu^{\prime }$ values, indicating that AAS itself is a relatively good microwave-absorbing matrix, as has been reported in the literature [38,39]. As the SS dosage is increasing, the peak frequencies of $\varepsilon^{\prime }$ and $\mu^{\prime }$ gradually shift toward the lower frequency range. This shift is mainly attributed to the Fe-bearing phases in SS that possess a high electromagnetic loss capability, such as α-Fe_2_O_3_ and Fe_3_O_4_. Nevertheless, the electromagnetic parameters of a material can only reflect its electromagnetic characteristics within a certain frequency range; achieving satisfactory microwave absorption properties ultimately depends on the balance between impedance matching and loss capacity.

#### 3.7.2. Microwave-Absorbing Properties

(1) Effects of SS dosages on microwave-absorbing properties

Based on the transmission line theory [38], the RL of AAS was calculated and evaluated using Equations (4) and (5). As can be seen from the above formulas, the calculation of RL is primarily based on transmission line theory, and the impedance matching condition has already been inherently incorporated into the calculation. A lower RL value suggests better microwave-absorbing properties, and in engineering practice, the frequency range where RL ≤ −10 dB is commonly regarded as the effective bandwidth.

(4) $$Z_{\mathrm{in}}=Z_{0}\sqrt{\mu_{r}{/\varepsilon }_{r}}\tanh(2\pi jdf\sqrt{\mu_{r}\varepsilon_{r}}/c)$$

(5) $$RL=20\log_{10}\left|Z_{\mathrm{in}}-Z_{0}\right|/\left|Z_{\mathrm{in}}+Z_{0}\right|$$

where *Z*_in_ is the input impedance of AAS, *Z*_0_ is the wave impedance of free space (377 Ω), and $\varepsilon_{r}$ and $\mu_{r}$ are the complex permittivity and complex permeability, respectively. *f* is the microwave frequency, *d* is the designed thickness of AAS, and c is the speed of light.

**Figure 13:**
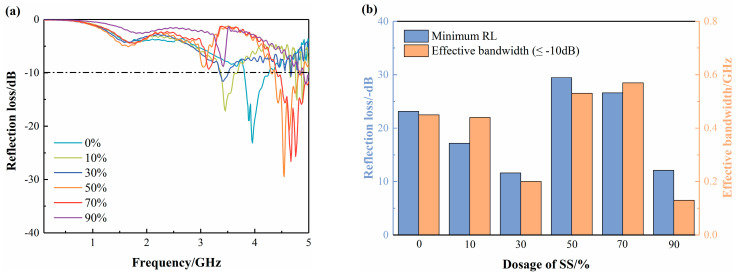
Microwave-absorbing properties of AAS with different SS dosages: (**a**) RL value; and (**b**) minimum RL and effective bandwidth.

According to Chinese standard JGJ/T 98-2010 [40], the design thickness of the AAS mortar was set as 20 mm, and their RL was calculated, as shown in Figure 13. Figure 13a presents the RL curves of AAS within 0.1–5 GHz. It can observed that the main absorption frequency band of AAS did not exhibit a linear variation with increasing SS dosage. Specifically, the main absorption peaks of B-S0, B-S10, and B-S30 were located in approximately in 3.5–4 GHz, while B-S50 and B-S70 were located in 4.5–5 GHz. According to the quarter-wavelength theory [41], the appearance of microwave absorption peaks is closely related to the electromagnetic parameters of each specimen at that frequency and to its thickness. For a given thickness, quarter-wavelength matching at the target frequency can only be achieved when the electromagnetic parameters satisfy specific conditions, thereby yielding optimal absorption performance. Figure 13b summarizes the minimum RL and effective bandwidth for each specimen at a thickness of 20 mm. Among them, B-S50 achieved the minimum RL value of −29.47 dB and an effective bandwidth of 0.53 GHz, whereas B-S70 presented a minimum RL of −26.60 GHz alongside an effective bandwidth of 0.57 GHz.

(2) Effects of absorber dosages on microwave-absorbing properties

Based on the performance indicators of AAS, including compressive strength, setting time, and drying shrinkage, the B-S50 was selected as the reference mixture. In previous studies [42,43], SF has been widely used in microwave-absorbing materials due to its good conductivity and ferromagnetism, contributing multiple loss mechanisms to absorbers. In this section, SF was used as a absorber, and its dosage was set as 0, 2, 4, 6, 8, and 10 wt.%. Finally, the effect of SF dosage on the microwave-absorbing properties of SS-containing AAS was investigated.

**Figure 14:**
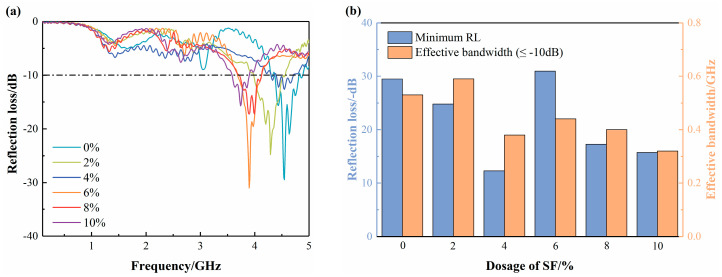
Microwave-absorbing properties of B-S50 with various SF dosages: (**a**) RL value; and (**b**) minimum RL and effective bandwidth.

Figure 14a shows the effects of SF dosages on the microwave-absorbing properties of SS-containing AAS with a 20 mm thickness, and the corresponding minimum RL and effective bandwidth are summarized in Figure 14b. The results indicate that SF did not improve the microwave-absorbing properties of AAS; the main absorption frequency range remained between 3.5 and 5 GHz. Specifically, B-S50-6 exhibited relatively better microwave-absorbing properties than the other samples, with a minimum RL of −30.97 dB and 0.44 GHz effective bandwidth. Theoretically, the incorporation of SF improves the dielectric loss and magnetic loss capacities of AAS due to its good conductivity and ferromagnetism [42]. The synergistic effect of these two mechanisms is generally beneficial for enhancing the microwave-absorbing properties. Specifically, as a highly conductive functional filler, SF can effectively enhance electrical conductivity. Under an external electromagnetic field, free electrons undergo directional drift within the fibers, generating conduction currents that dissipate electromagnetic wave energy. In addition, the ferromagnetic nature of SF gives rise to hysteresis loss and eddy current loss, which convert electromagnetic energy into heat energy under an alternating electromagnetic field. However, the observed counterintuitive result may stem from the following aspects: (1) the frequency range of the measurement targeted the low-frequency region (0.1–5 GHz), which does not cover the main frequency band where SF exerts its enhancement effect; (2) B-S50 already exhibited effective microwave-absorbing properties without any absorber addition, and the incorporation of SF may have disrupted the interfacial impedance matching; and (3) SF may not have formed a favorable match with the appropriate design thickness of AAS, leading to enhanced interface reflection.

(3) Effects of thicknesses on microwave-absorbing properties

**Figure 15:**
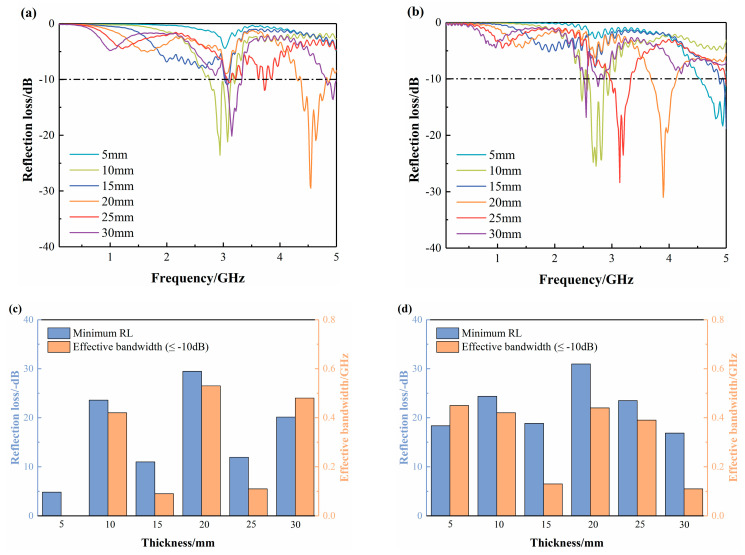
Microwave-absorbing properties of optimal sample with different thicknesses: (**a**) B-S50; (**b**) B-S50-6; and (**c**,**d**) minimum RL and effective bandwidth of B-S50 and B-S50-6.

Figure 15a and Figure 15b show the microwave-absorbing properties of B-S50 and B-S50-6 at 5–30 mm thickness, and the corresponding minimum RL and effective bandwidth were displayed in Figure 15c and Figure 15d, respectively. It can be observed that the minimum RL and effective bandwidth of B-S50 and B-S50-6 exhibit distinctly different thickness-dependent behaviors. Compared with B-S50, the B-S50-6 shows a lower RL and wider effective bandwidth across multiple thickness ranges, especially at thicknesses of 5 mm, 15 mm, and 25 mm. Specifically, the minimum RL is −18.35 dB with 0.45 GHz bandwidth at 5 mm thickness; these values are −18.84 dB and 0.13 GHz at 15 mm, and −23.50 dB and 0.39 GHz at 25 mm. Based on the above analysis, the effective microwave-absorbing properties of the SS-containing AAS without SF depends on specific thickness conditions. In contrast, after SF incorporation, the increased dielectric and magnetic loss capacities provided by SF enable effective microwave absorption across multiple thicknesses, thereby improving the flexibility of AAS material thickness design in practical engineering applications.

#### 3.7.3. Mechanisms of Microwave Absorption

**Figure 16:**
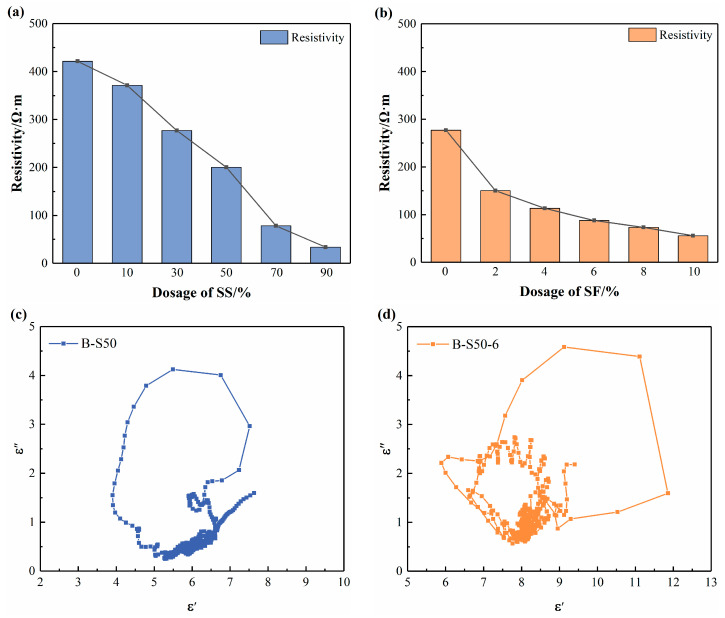
Resistivity and Cole–Cole plots: (**a**,**b**) Cole–Cole plots for B-S50 and B-S50-6; and (**c**,**d**) resistivity for B-S50 and B-S50-6.

The loss and attenuation mechanisms of microwaves in cementitious matrices are diverse. The resistivity measurement and Cole–Cole plot analysis are effective methods for elucidating the microwave attenuation mechanisms. A matrix with good conductivity can generate an induced magnetic field opposite to an external magnetic field, thereby canceling out the microwave energy. The increase in SS dosage significantly reduces the resistivity of AAS (see Figure 16a). As the SS content increased to 70% or even 90%, the matrix resistivity dropped sharply from 200.4 Ω·m (50% SS) to 78.3 and 33.8 Ω·m, respectively, with an inflection point observed in the resistivity curve, indicating that the minimum percolation threshold for forming a conductive network in the matrix had been essentially reached. This can be attributed to the large amount of Fe-bearing mineral phases (e.g., Fe_2_O_3_ and Fe_3_O_4_), which act as conductive particles and significantly promote the formation of the conductive network in AAS. Li et al. [38] investigated the microwave-absorbing properties of an SS-containing geopolymer, and the results showed that the significant promoting effect of SS on the microwave-absorbing properties of the geopolymer. On the other hand, SF can serve as a bridge connecting conductive particles and similarly reduces the resistivity to a great extent (see Figure 16b), establishing an electronic conduction mechanism in addition to the ionic conduction mechanism provided by SS. According to free electron theory [44], good electrical conductivity is an important prerequisite for dielectric loss. Therefore, both SS and SF collectively enhance the dielectric loss capacity of AAS.

The Debye model is commonly used to describe the polarization mechanisms of microwave-absorbing materials, and the semicircles observed in the Cole–Cole plots provide a basis for revealing the polarization mechanisms of AAS [45], as shown in Figure 16c,d. The incorporation of SF in SS-containing AAS led to significantly increased complexity of the Cole–Cole plots, and more semicircles were observed. These phenomena indicate that B-S50-6 underwent more Debye relaxation processes than B-S50, thus exhibiting distinct dielectric relaxation characteristics. Bai et al. [46] suggested that irregular curves in Cole–Cole plots are associated with polarization mechanisms such as dipole polarization and interfacial polarization. According to Maxwell’s electromagnetic theory [47], free charges at the interface between conductive fibers and the matrix undergo polarization relaxation under an alternating electric field. This may explain the irregular curves observed in the Cole–Cole plot of B-S50-6.

### 3.8. Discussion

This study proposes an eco-friendly design method for AAMs and systematically investigates their mechanical properties, workability, shrinkage characteristics, microstructure, and microwave-absorbing properties. SS, as a typical industrial solid waste, has attracted extensive attention from researchers [48,49]; however, its utilization in AAMs remains insufficiently comprehensive. This study found that the partial replacement of BFS with a low SS dosage does not significantly impair the mechanical properties of AAS (especially at 28 days), mainly because SS provides additional Ca^2+^ and OH^−^ for the BFS hydration, thereby promoting the formation of hydrates. Additionally, the fast-setting characteristic of AAS is highly detrimental to its practical engineering applications. Conventional AAS often fails to meet the workability requirements of actual projects in terms of initial and final setting times, necessitating the addition of various retarders to mitigate this fast-setting behavior [50]. However, using SS as a replacement precursor material in AAS substantially prolongs the setting time, offering a valuable design strategy for novel AAS. Furthermore, this study found that using SS as a 50% replacement precursor for BFS in AAS significantly alleviates the poor volume stability commonly observed in conventional AAS. Relevant studies have indicated that employing low-reactivity or inert precursors is an effective means of reducing the drying shrinkage of AAMs [30,38], and the conclusions drawn in this study are consistent with those findings.

**Figure 17:**
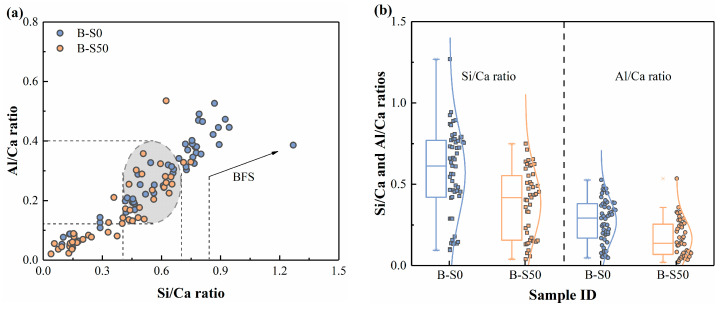
EDS point analysis: (**a**) Si/Ca vs. Al/Ca ratios; and (**b**) chemical compositions of gel hydrates.

A series of analytical methods, including XRD, TG/DTG, and SEM/EDS, were conducted to characterize the microstructure of SS-containing AAS in this study. The results show that C–(A)–S–H and N–A–S–H are the main hydrated gel products that constitute its microstructure. Ghafoor et al. [51] suggested that C–(A)–S–H and N–A–S–H are the hydrates in high- and low-calcium AAM systems, respectively, and that they can coexist in a blended system. In this study, the Ca content in SS is higher than that of BFS (see Table 1), providing additional Ca^2+^ sources for the AAM system. To clearly identify the effect of SS on the chemical composition of gel products in AAS, more than 40 EDS points (all targeting typical gel morphologies) were selected in B-S0 and B-S50 for elemental statistics, and their Si/Ca and Al/Ca ratios were calculated, as shown in Figure 17. The statistical results show that the incorporation of SS reduced the Si/Ca and Al/Ca ratios of the gel products. Shi et al. [52] believed that a decrease in the Si/Ca ratio leads to reduced crystallinity of the gel products, thereby affecting their micromechanical properties. Therefore, the chemical composition of the precursor materials (especially the relative contents of calcium, silicon, and aluminum) is a key factor that must be prioritized when designing the properties of AAMs.

Imparting good conductivity to a material is an important way to achieve microwave-absorbing properties [53]. However, the impedance design at the interface must be considered to avoid direct reflection of microwaves at the interface [54]. In interface impedance design, it is usually necessary to balance the four electromagnetic parameters of the material to maintain a balance between the input impedance and the impedance of free space. For AAS systems, the real part of the complex permittivity is generally in the range of approximately 5–7 (see Figure 12a). A high permittivity typically requires an increase in the material’s permeability to achieve good impedance matching. The large amount of Fe-bearing mineral phases provided by SS helps to increase the permeability of AAS, thereby optimizing the interface impedance performance. However, for many wave-absorbing materials used in building structures as reported in existing studies [55,56], the tested frequency band is almost exclusively 2–18 GHz, which makes it difficult to accurately evaluate the civilian core frequency bands such as FM broadcasting, 3G, 4G, and 5G communications. A previous work systematically investigated the microwave absorption functionality of geopolymer binders, and reported that the optimal sample achieved a minimum RL of −41.06 dB with a bandwidth of 0.50 GHz. These data are almost at the same level as those obtained in the present study, which fully demonstrates the performance advantages of the novel AAS proposed in this work [35]. On the other hand, SF, as a common reinforcing and toughening fiber, can not only alleviate the poor volume stability of AAS but also further improve its microwave-absorbing properties. Based on the findings in Section 3.7.3, it is believed that SF provides multiple microwave loss mechanisms for SS-containing AAS and promotes effective absorption of microwaves under thin thickness. Notably, the high electrical conductivity of SF brings about a Faraday cage effect, which introduces shielding characteristics. Therefore, achieving a balance between impedance matching and loss capacity is essential to obtain the desired microwave-absorbing properties. From a material perspective, the eco-friendly design method for AAMs proposed in this study only requires the combination of SS and SF to endow AAS with effective microwave absorption capability. From a design perspective, the incorporation of SF reduces the dependence of SS-containing AAS on a single design thickness, facilitating flexible material design to meet the requirements of various building components.

## 4. Conclusions

This study proposes an eco-friendly design method for AAMs. Based on the investigation of their mechanical properties, workability, and shrinkage characteristics, their microwave-absorbing properties were developed. The following conclusions can be drawn:

(1) The negative effect of SS on the compressive strength of AAS is mainly observed in the early-age hydration. The complexation reaction between BFS and the alkaline activator is the primary factor contributing to the early-age compressive strength, while the mineral phases in SS gradually participate in the hydration reaction at later stages. As the SS dosage is less than 50%, its adverse effect on strength is relatively limited.
(2) The incorporation of SS reduced the fluidity of AAS, but this reduction was significantly alleviated as the SS dosage exceeded 30%. Owing to the low reaction characteristic of SS, its presence lowered the early-age reaction rate of AAS, thereby markedly prolonging its setting time.
(3) With increasing SS dosage, the 28 d drying shrinkage of AAS first increased and then decreased. The main reason is that an appropriate SS dosage helps improve the pore structure and reduce the number of connected pores, thereby lowering the capillary pressure during water evaporation. Among them, B-S50 exhibited the lowest 28 d drying shrinkage of 778 με.
(4) The dissolution and hydration of SS provide additional Ca^2+^ and OH^−^ to the AAS system, thereby promoting the hydration of BFS. As the SS dosage increases, the amounts of C–(A)–S–H and N–A–S–H in the AAS system first increase and then decrease. As the SS dosage exceeds 50%, the hydration reaction rate decreases significantly, and the number of microcracks in AAS is markedly reduced.
(5) The Fe-bearing mineral phases in SS can serve as conductive fillers to significantly increase the conductivity of AAS, enhance dielectric loss, and achieve effective microwave-absorbing properties at a 20 mm thickness. SF not only further increases the conductivity but also provides additional electromagnetic loss mechanisms, reducing the thickness dependence of AAS and enabling effective microwave absorption across multiple thickness ranges.

## Figures and Tables

**Table 1:** Chemical composition of BFS and SS.

Composites	CaO	SiO_2_	Al_2_O_3_	Fe_2_O_3_	MgO	SO_3_	Na_2_O	MnO	K_2_O
BFS/wt.%	36.82	26.75	19.66	0.32	11.10	2.65	0.84	0.37	0.29
SS/wt.%	44.64	15.19	3.38	22.24	4.18	0.41	0.07	1.34	0.02

**Table 2:** Mix design of AAS mortars.

Sample ID	BFS/g	SS/g	Sand/g	Modified Water Glass/g	Water/g
B-S0	450	0	1350	90	157.5
B-S10	405	45	1350	90	157.5
B-S30	315	135	1350	90	157.5
B-S50	225	225	1350	90	157.5
B-S70	135	315	1350	90	157.5
B-S90	45	405	1350	90	157.5

**Table 3:** Qualitative results of hydrates in each specimen.

Item	Weight Loss/%			
	C–(A)–S–H/N–A–S–H		CaCO_3_	
	3 d	28 d	3 d	28 d
0%	4.81	6.61	4.62	5.29
10%	4.82	7.16	4.51	7.2
30%	5.43	6.77	4.34	5.84
50%	5.29	6.12	4.65	6.33
70%	4.19	6.25	4.16	5.49
90%	3.65	6.11	7.04	7.06

## Data Availability

The original contributions presented in this study are included in the article. Further inquiries can be directed to the corresponding author.
